# Pharmacokinetics in Zebrafish Embryos (ZFE) Following Immersion and Intrayolk Administration: A Fluorescence-Based Analysis

**DOI:** 10.3390/ph14060576

**Published:** 2021-06-16

**Authors:** Marlly Guarin, Annelii Ny, Noémie De Croze, Jan Maes, Marc Léonard, Pieter Annaert, Peter A. M. de Witte

**Affiliations:** 1 Laboratory for Molecular Biodiscovery, Department of Pharmaceutical and Pharmacological Sciences, University of Leuven, 3000 Leuven, Belgium; marlly.guarin@kuleuven.be (M.G.); annelii.ny@kuleuven.be (A.N.); jan.maes@kuleuven.be (J.M.); 2 L’Oréal Research & Innovation, 93600 Aulnay-sous-Bois, France; noemie.decroze@rd.loreal.com (N.D.C.); marc.leonard@rd.loreal.com (M.L.); 3 Drug Delivery and Disposition, Department of Pharmaceutical and Pharmacological Sciences, University of Leuven, 3000 Leuven, Belgium

**Keywords:** zebrafish embryo, fish embryo toxicity test (FET), pharmacokinetics, non-compartmental analysis, fluorescent compounds, exposure routes, false-negatives, absorption, distribution, computational biology

## Abstract

Zebrafish embryos (ZFE) have increasingly gained in popularity as a model to perform safety screenings of compounds. Although immersion of ZFE is the main route of exposure used, evidence shows that not all small molecules are equally absorbed, possibly resulting in false-negative readouts and incorrect conclusions. In this study, we compared the pharmacokinetics of seven fluorescent compounds with known physicochemical properties that were administered to two-cell stage embryos by immersion or by IY microinjection. Absorption and distribution of the dyes were followed at various timepoints up to 120 hpf by spatiotemporal fluorescence imaging. The concentration (10 µM) and dose (2 mg/kg) used were selected as quantities typically applied in preclinical experiments and zebrafish studies. The data show that in the case of a lipophilic compound (log D: 1.73) the immersion procedure resulted in an intrabody exposure which is similar or higher than that seen after the IY microinjection. In contrast, zero to low intrabody exposure was reached after immersion of the embryos with less lipophilic compounds. In the latter case IY microinjection, a technical procedure that can be easily automated, is highly recommended.

## 1. Introduction

Zebrafish embryos (ZFE) have increasingly gained in popularity as a model to perform safety screenings of compounds. Due to their small size and transparent bodies, which enable testing of compounds in 96–384 microwell plates and assessment of phenotypes using a dissecting microscope, the ZFE model combines the high throughput capability of in vitro systems with the physiological complexity of a whole vertebrate organism [1,2]. Moreover, this model adheres to the 3Rs principle of animal experimentation by providing refinement and partial replacement over higher animal models [1].

Efforts have been made to standardize and validate the use of ZFE in toxicity evaluations for regulatory purposes, the most common one being the fish embryo toxicity test (FET) defined in guideline 236 of the OECD (Organization for Economic Cooperation and Development). FET involves the use of zebrafish to determine the acute toxicity of chemicals during their embryonic stages by exposing newly fertilized eggs to test chemicals dissolved in their surrounding medium for 96 hpf [3]. Busquet and collaborators [4] evaluated the reproducibility of this zebrafish-based assay and identified it as being a robust screening method, in general providing acceptable repeatability and reproducibility rates with a coefficient of variation <30%.

However, in the case of very toxic, volatile, or poorly soluble chemicals, the coefficient variation was >30%. This outcome highlights the importance of compound’s physicochemical properties for the uptake and intrabody distribution of substances tested. In fact, although immersion of ZFE is the main route of exposure used, evidence shows that not all small compounds are equally absorbed by ZFE. Furthermore, the bioavailability of a compound may also vary depending on the developmental stage of the ZFE at the initiation of the experiment [5]. 

Actually, compounds face two barriers before being taken up by the ZFE, i.e., the chorion, a N-linked glycoprotein envelope pierced by circular pores of a diameter of 0.5–1.5 µm, and the vitelline bilayer membrane that surrounds directly the embryo and yolk [6]. The chorion is known to affect chemical uptake, depending on the molecule size, ionic charge, and the physicochemical properties of the molecule, among other parameters [7,8]. Consequently, embryos have been dechorionated before exposure to chemicals to improve the sensitivity towards toxicants of the zebrafish-based testing platforms [9].

Some pharmaceuticals seem however to pass readily the chorion and accumulate in the perivitelline space between the chorion and the vitelline membrane [7]. A study exposing embryos to hydrophilic dyes, showed that 90% of compound present in the egg was not localized within the embryo or chorion but rather distributed into the perivitelline space [10]. In contrast, lipophilic dyes were taken up readily [10]. These results show that the membrane and not the chorion can form an obstacle for intrabody exposure to some chemicals. A similar explanation has been cited to account for the lack of activity of cryoprotectants for fish eggs and embryos [11]. Whatever the reason, a relative lack of absorption might result in false-negative readouts and lead to incorrect conclusions in safety assessment evaluations. To avoid issues related to immersion, administration of compounds by parenteral exposure, i.e., intrayolk microinjection (IY), has been implemented in young embryos [12]. However, data demonstrating how compounds distribute in ZFE after IY delivery is lacking. Moreover, a detailed comparison with results using the same chemicals obtained by the immersion route, and relating the data with their physicochemical characteristics, is missing. 

In this study, we compared the pharmacokinetics of seven fluorescent compounds with known physicochemical properties that were administered to two-cell stage embryos by immersion or by IY microinjection. Absorption and distribution of the dyes were examined at various timepoints after administration by spatiotemporal fluorescence imaging. Relative exposure levels were first calculated using noncompartmental pharmacokinetic analysis and then related to some physicochemical descriptors of the compounds.

## 2. Results

### 2.1. Spatiotemporal Imaging Following Immersion and IY Microinjection

**Figure 1:**
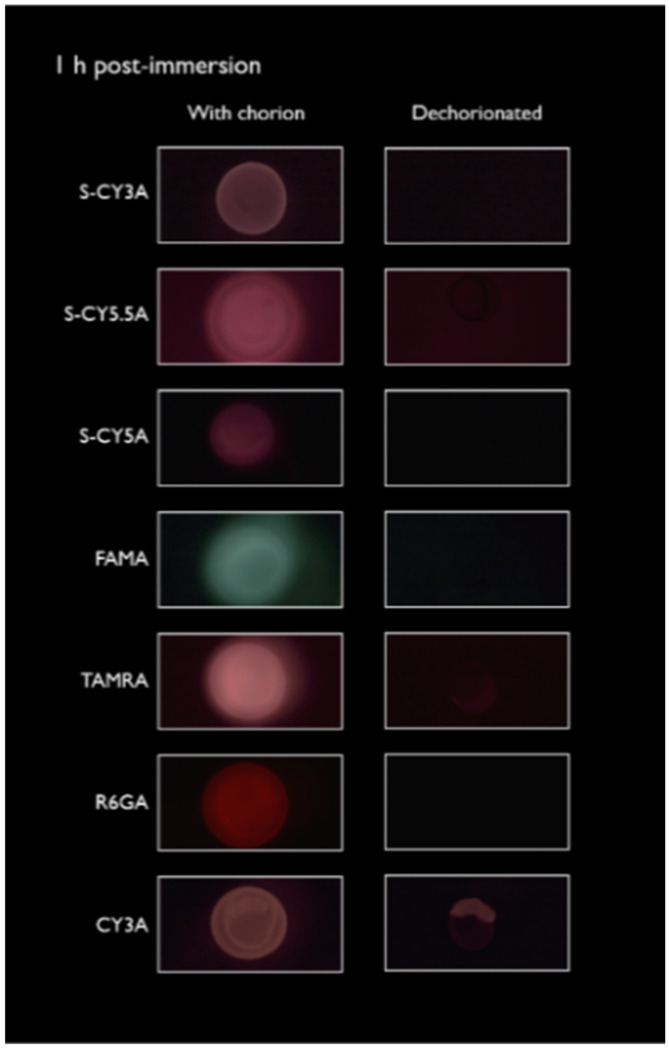
Representative fluorescent images of embryos exposed to the different fluorescent compounds by immersion (10 µM) for 1 h, before (left panel) and after dechorionation and rinsing (right panel).

Immersion: zebrafish embryos at the two-cell stage were immersed in a 10 µM solution of each of the seven fluorescent dyes for a maximum of 72 h. Afterwards the embryos were kept for another 48 h in Danieau’s medium in the absence of compound. At specific time points, the embryos were immobilized, and fluorescent pictures taken. As seen in Figure 1, embryos were highly fluorescent after 1 h immersion, but in most cases the fluorescence was lost after removal of the chorion and rinsing of the embryo, except in the case of CY3A. Longer incubations (24–72 h) resulted in a further intrabody accumulation of the compound, as also observed in the case of TAMRA. During the wash-out phase (72–120 h) some redistribution of the fluorescence could be observed.

**Figure 2:**
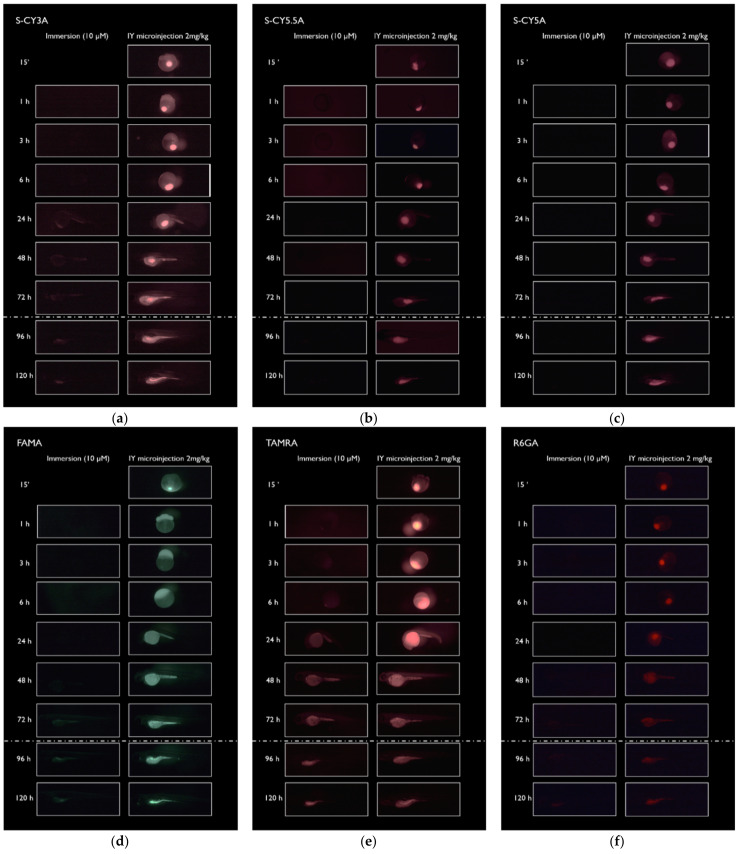

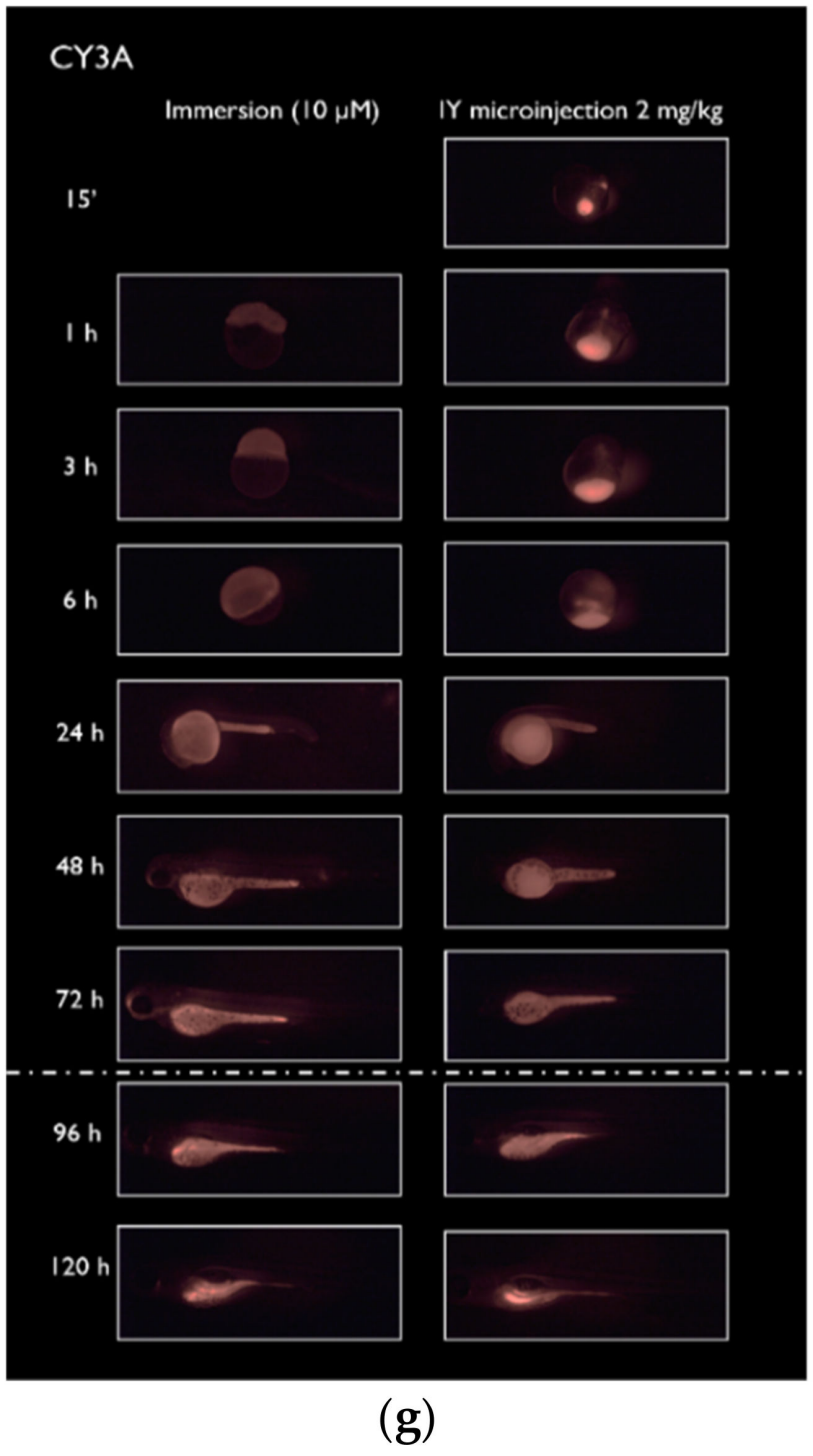
Representative fluorescent images showing the spatiotemporal distribution of the fluorescent dyes in the embryos after immersion (10 µM) and microinjection (2 mg/kg) for several periods of time. (**a**) animals treated with S-CY3A; (**b**) S-CY5.5A; (**c**) S-CY5A; (**d**) FAMA; (**e**) TAMRA; (**f**) R6GA; (**g**) CY3A. The dash dotted line represents the hatching time and the change of zebrafish medium. 15′ = 0.25 h.

IY microinjection: each of the compounds (2 mg/kg) was administered in zebrafish at the two-cell stage by microinjection in the yolk sac. In some embryos we could observe a flow of the dye from the injection site towards the proliferating embryonal cells during the first hours post injection (Supplementary Figure S1). However, a significant part of the compounds stayed localized to the microinjection site, especially in the case of dyes with a low Log D value, i.e., S-CY3A, S-CY5.5A and S-CY5A. At later time points, the fluorescent molecules distributed in the yolk area and also in the rest of the body, particularly in the 48–72 h period (Figure 2). Of interest, we also noticed an accumulation of S-CY3A, FAMA, TAMRA, R6GA, and CY3A in the gastrointestinal system after 96 hpf, and an excretion of some compounds by means of the cloaca that is functional at this developmental stage [13]. The spatiotemporal distribution of the fluorescent dyes in the embryos shows that the average fluorescence levels reached are substantially lower during immersion exposure than after microinjection, except for compound CY3A (Figure 2). 

### 2.2. Non-Compartmental Pharmacokinetic Analysis 

**Figure 3:**
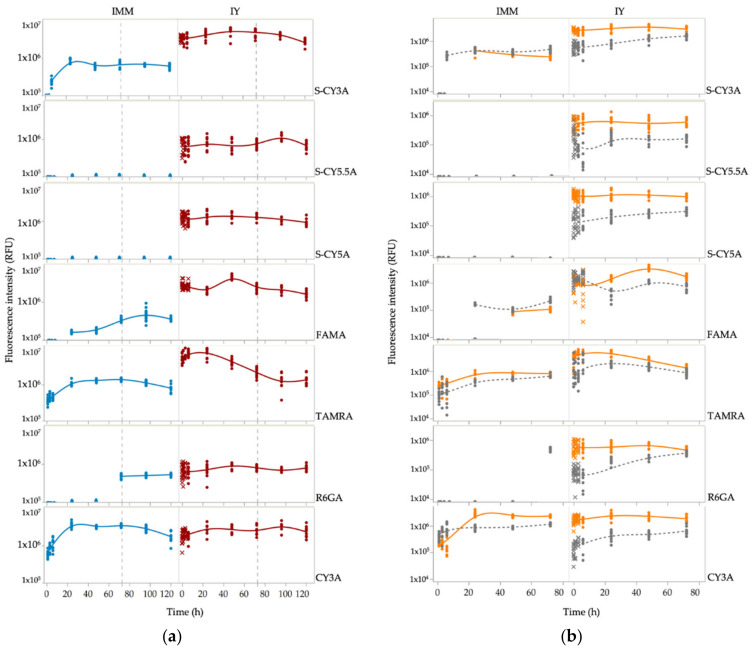
Semilogarithmic graphs of the fluorescence intensity (RFU) as a function of time, in the case of immersion (IMM) and intrayolk (IY) exposure routes (**a**) in the whole-body (from 0.25 to 120 h). The dashed line at 72 h indicates the start of the rinsing period. (**b**) in the yolk (orange line) and non-yolk parts (RoB, Rest of Body) (gray dash line) (from 0.25 to 72 h exposure). Excluded data points are marked as X symbols.

Next, we calculated the relative amount of fluorescent dye present in the embryos from 0.25 to 72 h post exposure by quantification of the integrated fluorescent intensity in delineated whole-body contours. Of notice, the maximal fluorescence intensity was not recorded immediately after microinjection, probably by temporal formation of aggregates that show no fluorescence during early time points [14]. These early observations were therefore censored per compound and excluded from the dataset. The semilogarithmic graphs in Figure 3a show that only in the case of CY3A the immersion and intrayolk microinjection procedure resulted in a similar exposure profile.

In addition, as the yolk makes up a significant portion of the zebrafish embryo during the first stages of development and is known to retain small molecules due to its high content of cholesterol and triacylglycerol [15], we also assessed the amount of fluorescent dye present in the yolk and in the rest of the body (RoB, i.e., non-yolk parts). We only measured up to 72 hpf as the yolk part becomes difficult to discern from the rest of the body in later periods. Figure 3b shows that after IY microinjections the amount of fluorescent dye present in the yolk was higher than in the RoB parts for all compounds during the whole time. In the case of immersions, the accumulation of the fluorescent dyes in yolk and RoB was less divergent.

We then assessed by non-compartmental analysis the area from 0.25 to 72 h post exposure under the concentration–time curve (AUC_0.25–72h_) (Table 1) that represents well the relative tissue exposure to fluorescent compound across time. These absolute fluorescence intensities however cannot be comscriptpared across compounds as they depend critically on the respective quantum yields of the compounds and the device settings. To be able to compare the PK behavior of the compounds following different administration routes, we calculated the fluorescence-independent pharmacokinetic parameter Relative Exposure (RE_10/2/72h_), i.e., the ratio of AUC_0.25–72h_ immersion (10 µM) to AUC_0.25–72h_ IY microinjection (2 mg/kg). Separate RE values were calculated based on the whole body (WB), rest-of-body (RoB) and yolk fluorescence measurements (Table 2).

The results show that the RE-WB_10/2/72h_ was low (0.05–0.19) for most compounds, except for CY3A (RE-WB_10/2/72h_: 1.11). A similar outcome was obtained for RE-RoB_10/2/72h_, although S-CY3A and CY3A displayed values that were two-fold as high as the corresponding RE-WB_10/2/72h_ results. The data indicate that tissue exposure was 3- to 9-fold higher for most compounds as a function of time when administered by microinjection than after immersion, except in the case of CY3A (i.e., two-fold higher after immersion). The RE-Yolk_10/2/72h_ values are low to very low for most compounds, except for Cy3A, demonstrating that non-lipophilic compounds are not readily taken up by yolk after immersion. 

Based on the AUC_0.25–72h_ results, we further calculated the Relative Distribution (RD) after microinjection, i.e., the ratio of AUC_RoB_ to AUC_WB_ after microinjection of the compounds into the yolk (2 mg/kg). The data show that all microinjections resulted in rather low RD values, ranging from 0.17 to 0.32 (Table 2), indicating that only a partial amount of the dose injected was redistributed during the first 72 h to the non-yolk parts of the ZFE.

### 2.3. QSPkR Analysis

**Figure 4:**
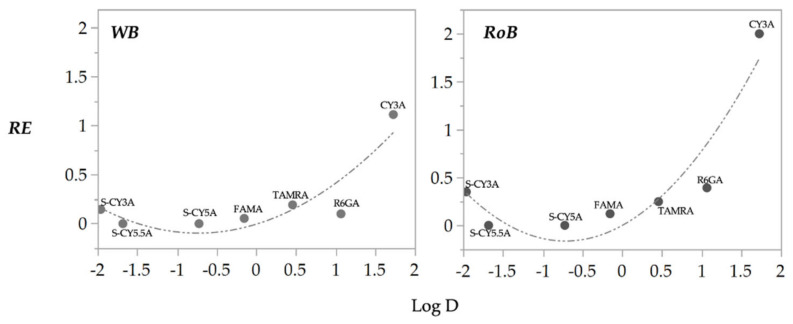
Relationship between observed RE and Log D for the WB (**left**) and the RoB (**right**) of the 7 fluorescent compounds. The dashed line is line of fit.

Next, we performed a QSPkR (Quantitative Structure-Pharmacokinetic Relationship) analysis to correlate calculated RE values with a set of molecular descriptors of the fluorescent dyes used (Table 3). Through multiple regression modelling (MLR) we identified a parabolic relationship between RE_WB_ and RE_RoB_, and Log D (R^2^ 0.81, RMSE 0.213, *p* < 0.037; R^2^ 0.82, RMSE 0.299, P 0.015; respectively) (Figure 4). In the case of RE_Yolk_, the outcome also included other descriptors, i.e., TPSA, MR and MR (R^2^ 0.99, RMSE 0.043, *p* 0.011). The RMSE values of the RE_WB_ and RE_RoB_ models are in the range of observed RE values for compounds with Log D values below 1. As illustrated in Figure 4, this is consistent with comparable RE values for 6/7 compounds tested in the range −2 < Log D < 1. Nevertheless, the models clearly predict substantial increase in these RE values for compounds with Log D values above the cut-off of 1.5. In addition, adequate correlations between the RE values and Log D values are also reflected by the corresponding R^2^ and *p*-values (see Table 3).

## 3. Discussion

In this study we compared the pharmacokinetics of seven photostable fluorescent compounds encompassing Log D values from −1.96 to 1.73 in zebrafish embryos after immersion or microinjection. The concentration (10 µM) and dose (2 mg/kg) used were selected as quantities typically applied in preclinical experiments and zebrafish studies [16]. Moreover, these exposures did not induce any sign of toxicity in the embryos while resulting in quantifiable fluorescence levels in the organism.

The spatiotemporal imaging after one hour of immersion followed by dechorionation of the zebrafish embryos showed that most of the fluorescent compounds were associated with the chorion or accumulated in the perivitelline space. However, this observation does not reflect a real uptake of the compounds by the developing embryo, possibly resulting in false-negative results when toxicity or teratogenicity testing is performed. Only in the case of the most lipophilic compound (Cy3A, Log D: 1.73) could a fast intrabody accumulation be observed, implying that the compound readily passed the vitelline membrane and penetrated the embryonic cells.

The images further show that, especially when compared to the fluorescence observed after IY microinjections, most of the less lipophilic compounds were not taken up well by the embryos, even after longer exposure periods (up to 72 h). Our results therefore are in line with a previous study that concluded that lipophilic dyes are better absorbed from the aqueous environment than less lipophilic ones during zebrafish embryo development [10].

Of interest, immediately after the IY microinjection a flow of some fluorescent dyes could be observed in the direction of the embryonic cells. This process is probably driven by the dynamic ooplasmic streaming that takes place in the early embryonic development [17]. However, a major part of the dyes stayed localized to the microinjection site that was redistributed over the non-yolk body parts during the following hours. The relative distribution (RD) data that compare the amount of fluorescence in the non-yolk parts with the total fluorescence present over a period of 72 h post-injection, indicate that about 20–30% of compound entered the rest of the embryonal body. This outcome therefore reveals that molecules are released from the dense amphiphilic environment of the yolk independent of the lipophilicity, although only slowly. The data are somewhat in contrast to the results obtained in our previous study, where a lack of intrabody distribution was observed in most cases after injecting the fluorescent compounds into the yolk of 3 dpf eleuthero-embryos [15,16]. So, although a side-by-side comparison is lacking, it appears that administering compounds by microinjection at 0 dpf or 3 dpf disperses the compounds differently into the rest of the body. Clearly, more work is needed to better understand the mechanisms underlying this phenomenon. We anticipate particularly that the fast consumption of the yolk during the first days with a dynamic supply of protein, lipids, and micronutrients to the rest of the body [15], can substantially influence the biodistribution of compounds after microinjection.

Although the amount of compound microinjected in the yolk redistributed only partially, the Relative Exposure (RE_10/2/72h_) data clearly prove that drug exposures in the whole body (WB), Rest-of-Body (RoB) and yolk, were substantially higher after microinjection than after the immersion procedure for most compounds. Significantly, the outcome was different for the most lipophilic compound CY3A that reached a RE-RoB value of 1.99 after 72 h, showing that the non-yolk body tissues and organs were more exposed to the compound after immersion than after intrayolk microinjection. 

Non-compartmental pharmacokinetic analysis was also performed, followed by a *QSPkR* assessment, revealing that *RE*_WB10/2/72h_ and the *RE*_RoB10/2/72h_ were consistently estimated by the lipophilicity level of the compounds, whereas the estimation of RE_Yolk10/2/72h_ required additional molecular descriptors, i.e., TPSA, MR and the interaction of MR with the lipophilicity level of the compounds. Compound lipophilicity has been previously identified as the most critical physicochemical property for the absorption of molecules in zebrafish [16,18,19] as also found in mammals [20]. Moreover, the identification of TPSA, MR, Log D as properties that explain the relative amount of compound in the zebrafish yolk can be related to the lipidic character and the rheological properties of the yolk. TPSA and MR are molecular descriptors containing information about the compounds’ polarizability, thus accurately taking into account electronic parameters that have an effect on chemical–biological interactions [19,20,21,22,23,24,25,26]. This understanding aligns with the biochemical composition of the yolk, which is different from RoB [15].

## 4. Materials and Methods

### 4.1. Zebrafish

Adult wildtype (AB) zebrafish (Danio rerio) were maintained at 28.5 °C on a 14/10 h light/dark cycle according to standard zebrafish aquaculture conditions [24]. The fish were fed with a commercial fish diet twice a day and live food (Artemia salina) once a day. The embryos were collected from natural spawning and raised in Danieau’s solution 0.3× (17 mM NaCl, 0.2 mM KCl, 0.18 mM Ca(NO_3_)_2_, 0.12 mM MgSO_4_ and 1.5 mM HEPES buffer pH 7.1–7.3) [25].

### 4.2. Fluorescent Compounds

Fluorescent compounds were acquired from Lumiprobe (Hannover, Germany): alkyne cyanine-based dyes: S-CY3A (CAS No. A13B0), S-CY5.5A (CAS No. A73B0), S-CY5A (CAS No. A33B0) CY3A (CAS No. A10B0), and alkyne xanthene-based dyes: FAMA (CAS No. A41B0), TAMRA (CAS No. A71B0) and R6GA (CAS No. A52B0). These compounds represent a wide range of Log D values that were determined experimentally before [16], as listed in Table 4. This table also includes molecular descriptors of the compounds as calculated by the SwissADME platform [26]. All compounds were dissolved in DMSO (99.9%) and frozen as 10 mM stock solutions at −20 °C.

### 4.3. Fluorescent Compound Treatments

Immersion treatment: zebrafish embryos at the two-cell stage were immersed in Danieau’s medium containing the compound. A compound concentration of 10 µM and DMSO concentration of 0.1% (v/v) in a volume of 5 mL was used per well (6-well-plates). At 72 h, the zebrafish medium containing the compound was removed, and the animals rinsed 3 × with Danieau’s medium. Next, the embryos were kept for another 48 h in Danieau’s medium supplemented with DMSO (0.1%, v/v) in the absence of compound. In the case of control experiments, the embryos were exposed to Danieau’s medium supplemented with DMSO (0.1%, v/v).

Intrayolk microinjection (IY): zebrafish embryos at the two-cell stage were positioned in a Petri dish at room temperature. IY microinjection was performed according to Rosen (2009) [12], using glass needles fitted to a micromanipulator (MM-33) connected to a gas pressure microinjector (Eppendorf Femtojet AG, Hamburg, Germany). The glass capillaries (W/FIL 1.0MM 4 in TW 100F-4) were pulled (Sutter Instrument CO. Model P-87 Cat N B100-58-15 Filament: FB330B–B320B, Novato, CA, USA) using program 5 (Heat 829, Pull 158, Vel 100, Time 150). Needles were filled with compounds dissolved in the vehicle (DMSO/saline (1:1)), placed under the microscope, and using forceps, the tip was cut off in a manner to allow for a consistent volume to be injected. Afterwards, the embryos were transferred to 6-well plates. Control embryos were exposed to vehicle only.

### 4.4. Spatiotemporal Fluorescence Imaging

Animals were kept in dark conditions in an incubator at 28.5 °C for defined time-periods (0.25, 1, 3, 6, 24, 48, 72, 96, and 120 h) before image analysis in a dark room. To acquire images, a Leica MZ10F fluorescent stereomicroscope (Leica Microsystems Inc., Buffalo Grove, IL, USA) with a 4.0× planapochromatic objective (10447243) was used, equipped with a Digital Color Camera Leica DFC310 FX (Software LAS 4.13, Leica Microsystems Inc., Buffalo Grove, IL, USA). Filter sets were GFP 10446222 in the case of compound FAMA, dSRED 10447079 in the cases of S-CY3A, CY3A and TAMRA, and CY5 10446366 in the cases of S-CY5.5A and S-CY5A. Previous to fluorescence imaging, the embryos were dechorionated (up to 72 hpf) and immobilized by hypothermia, rinsed three times with Danieau’s medium, and positioned latero-lateral (right lateral recumbency) on a single cavity glass slide and covered by a drop of agarose (0.1%). Then, using MetaMorph (Microscopy Automation and Image Analysis Software V.7.8.00, Molecular Devices, LLC., San Jose, CA, USA) and by manual delineation of the whole-body (WB) (up to 120 h) and yolk sac contours (up to 72 h) of the zebrafish embryos the fluorescence in the selected area was quantified as integrated fluorescence intensity (RFU) [16]. The RFU values of the non-yolk compartment (i.e., RoB: rest of body) was assessed by subtracting the yolk results from the corresponding integrated fluorescence intensities found in the WB.

### 4.5. Non-Compartmental Pharmacokinetic Analysis

In order to determine the degree of tissue exposure following administration of the fluorescent compounds, we calculated the AUC as the pharmacokinetic parameter of the non-compartmental analysis. The area under a plot of the fluorescence intensity (*RFU_T_*) versus time is referred to as the area under the (zero moment) curve *AUC. RFU_T_* represents the relative amount of compound present in the ZFE by measuring the integrated fluorescence intensity of delineated contours in fluorescence images (Figure 2). The AUC was then determined by summing the incremental area of subsequent trapezoids [27]. Therefore, the linear trapezoidal method for calculation of the AUC is (Equation (1)):

(1) $$AUC_{0.25}^{72h}=\overset{n}{\underset{i=0.25h}{\sum }}\frac{RFU_{Ti}+RFU_{Ti+1}}{2}.\;\Delta t,$$

where RoB*t* = *t*_0.25′+1_ − *t*_0.25_ and *t*_72h_ denotes the time of the last measured *RFU_T_*. In some cases, observations were censored and excluded from the analysis, due to possible fluorescence quenching effect of compounds that remains at the site of the intrayolk microinjection.

We further calculated the Relative Exposure (*RE*_10/2_) by comparing the *AUC*_0–120h_ after immersion (10 µM) with the *AUC*_0–120h_ after IY microinjection (2 mg/kg), as defined in Equation (2) [16]:

(2) $$RE_{10/2/h}=\frac{AUC_{Imm}}{AUC_{Inj}}.$$

### 4.6. Statistical Analysis

The non-compartmental and the QSPkR analyses were performed using JMP (Version 15.1. SAS Institute Inc., Cary, NC, USA). We ran the QSPkR analysis identifying the association among *RE*, with the experimental LogD value and some molecular descriptors applying a multiple linear regression analysis.

## 5. Conclusions

In conclusion, by using a fluorescence-based approach in this study, we show that a 72 h-long immersion of embryos starting at a two-cell stage results in an intrabody exposure which is similar or higher than that seen after a 2 mg/kg intrayolk microinjection, at least in the case of a lipophilic compound (log D: 1.73). In contrast, zero to low intrabody exposure was reached after immersion of the embryos with less lipophilic compounds, possibly resulting in a false-negative outcome in screening programs. In the latter case IY microinjection, a technical procedure that can be easily automated, is highly recommended [12,22,23]. Alternatively, higher immersion concentrations than the one used in this study could possibly be deployed in order to increase intrabody exposure to compounds. Future studies should consider examining the relationship between immersion concentrations and the relative uptake in ZFE, essential information that is presently missing in literature.

## Figures and Tables

**Table 1:** AUC_0.25–72h_ values calculated following immersion (10 µM) and intrayolk microinjection (2 mg/kg) from 0.25 to 72 h for the whole-body (WB), yolk and RoB parts. AUC_0.25–72h_ values expressed as RFU × 10^7^.

Compound	*AUC-WB*		*AUC-Yolk*		*AUC-RoB*	
	IMM	IY	IMM	IY	IMM	IY
S-CY3A	4.56	31.4	1.89	23.8	2.67	7.65
S-CY5.5A	0	5.45	0	4.42	0	1.02
S-CY5A	0	9.49	0	7.82	0	1.67
FAMA	1.17	27.8	0.36	14.9	0.84	6.93
TAMRA	7.76	40.4	4.98	29.1	2.78	11.2
R6GA	0.58	5.76	0	4.29	0.58	1.48
CY3A	20.9	18.9	14.6	15.7	6.41	3.21

**Table 2:** Relative exposure (*RE*) values (*WB*: whole body, *RoB*: rest of body) in the case of immersion and intrayolk microinjection and relative distribution ratio (*RD*) after microinjection.

Compound	*RE-WB*	*RE-RoB*	*RE-Yolk*	*RD* (IY)
S-CY3A	0.15	0.35	0.08	0.24
S-CY5.5A	0	0	0	0.19
S-CY5A	0	0	0	0.18
FAMA	0.05	0.12	0.02	0.32
TAMRA	0.19	0.25	0.17	0.28
R6GA	0.10	0.39	0	0.26
CY3A	1.11	1.99	0.93	0.17

**Table 3:** *QSPkR* analysis results. MLR of the *RE* and molecular descriptors as explanatory variable of the models. Only statistically significant models are shown. adj: R^2^ adjusted. RMSE: root mean square error. *p*-Value (<0.05).

PK. Parameter	Model	R^2^ adj	RMSE	*p*-Value
RE_WB10/2/72h_	=−0.011 + 0.187(Log D) + (Log D + 0.179)^2^ × 0.169	0.81	0.213	0.037
RE_RoB10/2/72h_	=−0.013 + 0.340(Log D) + (Log D + 0.179)^2^ × 0.320	0.82	0.299	0.015
RE_Yolk10/2/72h_	=−1.473 + 0.313(Log D) + 0.006(TPSA) + 0.009(MR) + (Log D + 0.179) (MR − 166.591) × 0.015	0.99	0.043	0.011

**Table 4:** Molecular descriptors of the fluorescent compounds and the experimentally determined Log D values.

Compound NO.	S-Cyanine 3 (S-CY3A) 1	S-Cyanine 5.5 (S-CY5.5A) 2	S-Cyanine 5A (S-CY5A) 3	Fam A, 5-Isomer (FAMA) 4	Tamra A 5-Isomer (TAMRA) 5	Rhodamine 6g 6-Isomer (R6GA) 6	Cyanine 3 (CY3A) 7
MW g/mol	691.9	1054.36	547.79	413.38	467.52	462.6	530.14
Rotor	13	18	11	3	6	7	10
HBA	7	13	1	6	4	2	1
HBD	1	1	0	3	1	1	1
MR	180.42	241.21	185.18	109.52	135.27	144.59	169.95
TPSA Å^2^	152.68	256.18	23.32	105.09	88.62	38.33	35.35
Log D	−1.96	−1.68	−0.72	−0.14	0.46	1.07	1.73

## Data Availability

The data supporting reported results are digitally archived and can be obtained from the corresponding author upon reasonable request.

## Supplementary Materials

The following are available online at https://www.mdpi.com/article/10.3390/ph14060576/s1, Figure S1: Representative images of embryos showing the flow of some fluorescent dyes from the yolk (bottom) to the proliferating embryonic cells (top) after intrayolk microinjections (2 mg/kg) in compounds S-CY3A (1 h), FAMA (0.25 h), TAMRA (1 h), and CY3A (1 h).
