# Supplementary material for: Pharmacokinetics in Zebrafish Embryos (ZFE) Following Immersion and Intrayolk Administration: A Fluorescence-Based Analysis
Source: Pharmaceuticals (Basel). 2021 Jun 16;14(6):576. doi: 10.3390/ph14060576 (PMC8234359; doi:10.3390/ph14060576)
Supplement: Supplementary file 1 [file pharmaceuticals-14-00576-s001.zip › pharmaceuticals-1258533-SI.pdf]

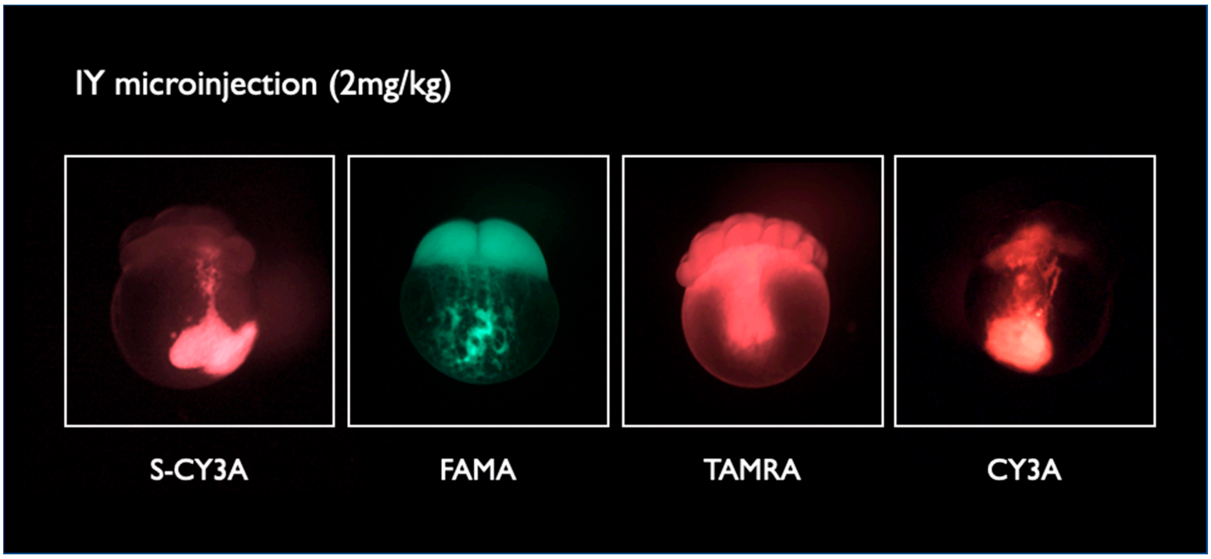

Supplementary Material Figure S1. Representative images of embryos showing the flow of some fluorescent dyes from the yolk (bottom) to the proliferating embryonic cells (top) after intrayolk microinjections (2 mg/kg) in compounds S-CY3A (1 h), FAMA (0.25 h) , TAMRA (1 h), and CY3A (1 h).
